# Antiarrhythmic Drug Therapy in Cardiomyopathies: A Comparative Synthesis of Guidelines and Consensus Documents

**DOI:** 10.1007/s11886-026-02405-0

**Published:** 2026-08-27

**Authors:** Andrea Palermi, Massimo Di Marco, Silvio Saraullo, Roberta Magnano, Laura Pezzi, Davide Rossi, Giulia Renda, Sabina Gallina, Gianfranco Sinagra, Marco Merlo

**Affiliations:** 1 Department of Neuroscience, Imaging and Clinical Sciences, G. D’Annunzio University of Chieti-Pescara, Via L. Polacchi 11, 66100 Chieti, Italy; 2 Cardiology and ICCU Department, Santo Spirito Hospital, Pescara, 65124 Italy; 3 University Cardiology Division, Heart Department, SS Annunziata Polyclinic University Hospital, Chieti, 66100 Italy; 4 Centre for Diagnosis and Treatment of Cardiomyopathies, Cardiovascular Department, Azienda Sanitaria Universitaria Giuliano-Isontina (ASUGI), European Reference Network for Rare, Low Prevalence and Complex Diseases of the Heart (ERN GUARD-Heart), Trieste, Italy; 5 University of Trieste, Trieste, Italy

**Keywords:** Antiarrhythmic drugs, Cardiomyopathies, Hypertrophic cardiomyopathy, Arrhythmogenic cardiomyopathy, Dilated cardiomyopathy, Ventricular arrhythmias

## Abstract

**Purpose of Review:**

Antiarrhythmic drug therapy in cardiomyopathies remains challenging because arrhythmic risk, drug efficacy, and pro-arrhythmic vulnerability vary substantially across phenotypes. This review summarizes contemporary guideline and consensus recommendations on antiarrhythmic drug use in hypertrophic, dilated, non-dilated left ventricular, and arrhythmogenic cardiomyopathies.

**Recent Findings:**

Recent guidelines increasingly emphasize phenotype-oriented arrhythmia management, but specific recommendations for antiarrhythmic drug therapy remain fragmented. Beta-blockers and amiodarone are the most consistently recommended agents in structural heart disease, whereas class I drugs are generally restricted. Evidence is strongest for hypertrophic and arrhythmogenic cardiomyopathies, while recommendations for dilated and non-dilated left ventricular cardiomyopathies are largely extrapolated from broader heart failure populations.

**Summary:**

Antiarrhythmic drugs are mainly used to reduce arrhythmic burden, symptoms, and implantable cardioverter-defibrillator therapies rather than to improve survival. Future studies should define phenotype-specific strategies based on ventricular function, myocardial scar, genotype, and clinical context.

## Introduction

Cardiomyopathies represent a heterogeneous group of myocardial disorders characterized by structural and functional abnormalities, often occurring on a genetic background that influences disease phenotype, arrhythmic substrate, and clinical progression. Across the spectrum of cardiomyopathies, including hypertrophic, arrhythmogenic, dilated and non-dilated left ventricular forms, arrhythmias are highly prevalent and contribute significantly to morbidity and mortality, ranging from symptomatic atrial fibrillation to life-threatening ventricular tachyarrhythmias and sudden cardiac death [1].

The arrhythmic risk in cardiomyopathies is not uniform but varies according to the underlying phenotype, genetic substrate, and disease stage. Ventricular arrhythmias represent the clinical hallmark of arrhythmogenic cardiomyopathy [2] and are frequently observed in dilated cardiomyopathy, where myocardial fibrosis, scar-related conduction heterogeneity, and adverse ventricular remodeling create a substrate for re-entry and contribute substantially to sudden cardiac death [3]. Conversely, atrial fibrillation is particularly common in hypertrophic cardiomyopathy and in advanced cardiomyopathies, largely reflecting atrial enlargement, diastolic dysfunction, and increased filling pressures [4]. This marked heterogeneity complicates therapeutic decision-making and requires individualized management strategies [5]. AAD therapy remains a key component in the management of these patients, either as a primary strategy or in combination with device-based and interventional approaches. However, the use of AADs in cardiomyopathies is particularly challenging. In addition to their variable efficacy, many agents carry an intrinsic risk of pro-arrhythmia, which may be amplified in the presence of structural heart disease, myocardial fibrosis, and electrical instability. As a result, the therapeutic window is often narrow, and careful patient selection is essential [6].

Although several international guidelines and consensus documents provide recommendations on arrhythmia management, guidance on AAD use in cardiomyopathies remains fragmented across different documents and is not consistently addressed in a phenotype-specific manner. Therefore, the aim of this systematic review is to synthesize current guidelines and consensus recommendations on the use of AADs in cardiomyopathies, providing a clinically oriented overview and highlighting areas of agreement, discrepancies, and gaps in evidence.

## Materials and Methods

### Study Design and Search Strategy

This study was conducted as a systematic review and comparative synthesis of clinical practice guidelines and consensus statements addressing the use of AADs in cardiomyopathies. The review was performed in accordance with the Preferred Reporting Items for Systematic Reviews and Meta-Analyses (PRISMA) principles. A structured literature search was performed in PubMed to identify relevant documents published in English over the last 15 years (from 2011 to 2026). The search strategy combined terms related to cardiomyopathies (including arrhythmogenic cardiomyopathy, arrhythmogenic right ventricular cardiomyopathy, hypertrophic cardiomyopathy, dilated cardiomyopathy, non-dilated left ventricular cardiomyopathy, and restrictive cardiomyopathy), antiarrhythmic therapy, and clinical practice guidelines or consensus documents.

In addition to database searching, a manual search of major scientific society websites, including the European Society of Cardiology (ESC), European Heart Rhythm Association (EHRA), Heart Rhythm Society (HRS), and American Heart Association/American College of Cardiology (AHA/ACC), was performed to identify additional relevant guidelines and consensus statements consistent with the study scope.

Furthermore, selected guidelines addressing specific clinical scenarios not directly captured by the primary search strategy, such as pregnancy, were considered for contextual interpretation but were not included in the systematic selection process.

### Study Selection

Titles and abstracts were independently screened by two reviewers in a blinded manner using the Rayyan web-based platform. Discrepancies were resolved by consensus. Records were eligible for inclusion if they were official clinical practice guidelines or consensus statements providing recommendations on the management of cardiomyopathies with specific reference to AADs. Reviews, original studies, meta-analyses, case reports, and documents not providing specific recommendations on antiarrhythmic therapy were excluded.

### Data Extraction and Synthesis

Full-text articles were assessed for eligibility, and relevant data were extracted, including issuing society, year of publication, scope, and recommendations related to AAD use. Given the heterogeneity of the available evidence and the nature of the included documents, a qualitative narrative synthesis was performed to compare recommendations across different guidelines and consensus statements.

### Study Flow

**Fig. 1:**
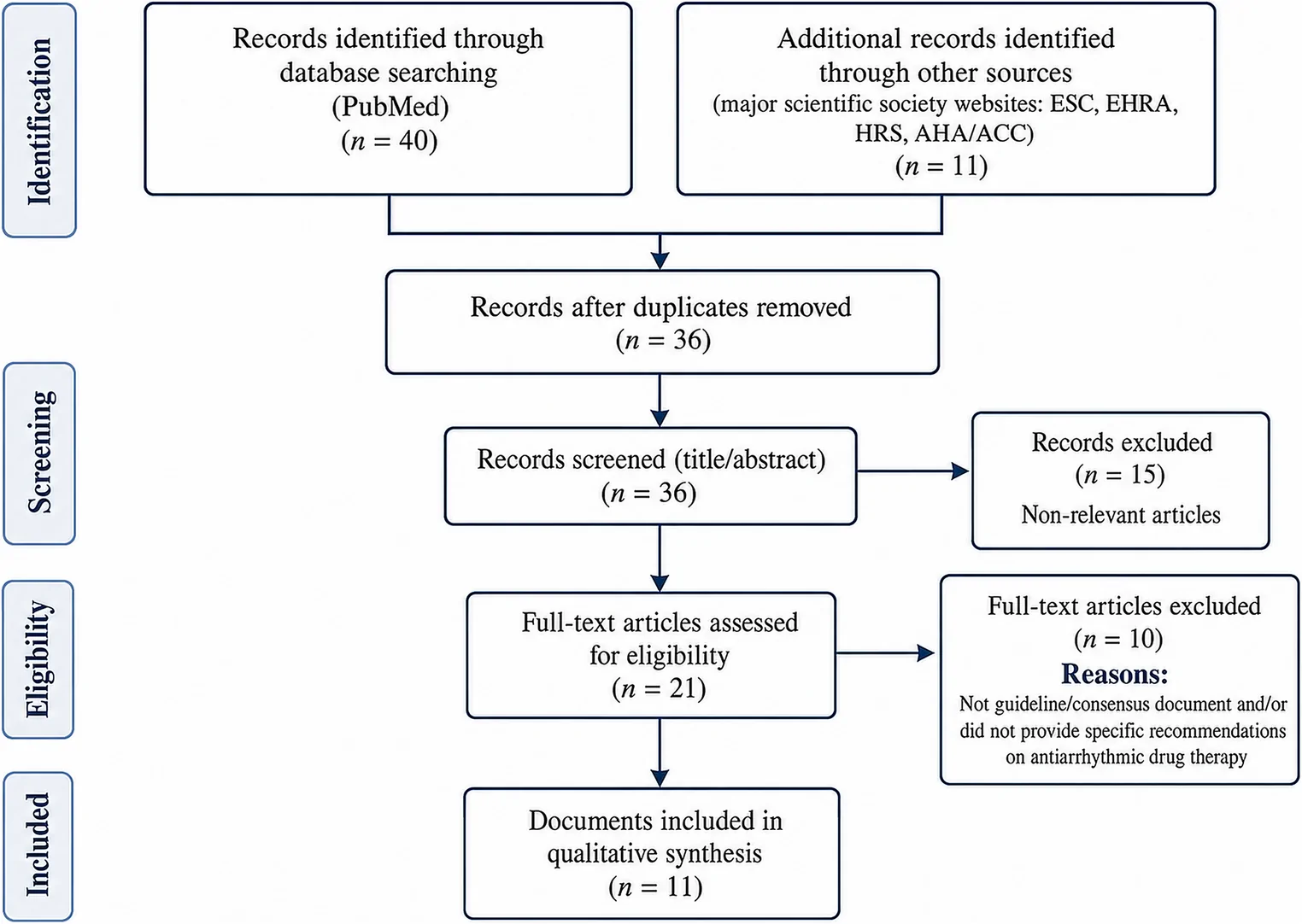
PRISMA flow diagram of study selection. Flow diagram summarizing record identification, screening, eligibility assessment, and final inclusion of clinical practice guidelines and consensus documents. Overall, 51 records were identified, 36 were screened after duplicate removal, 21 underwent full-text assessment, and 11 documents were included in the qualitative synthesis. Created in BioRender. Palermi, A. (2026) https://BioRender.com/sun63ai

A total of 51 records were identified, including 40 from PubMed and 11 from manual searches of major scientific society websites. After removal of 15 duplicates, 36 records were screened based on title and abstract. Of these, 15 were excluded, leaving 21 documents for full-text assessment. Following full-text review, 10 documents were excluded because they were not guideline or consensus documents and/or did not provide specific recommendations on AAD therapy. Overall, 11 documents were included in the final analysis. The complete study selection process is summarized in the PRISMA flow diagram shown in Fig. 1.

## Results

### Overview

A total of 11 clinical practice guidelines and consensus documents from major international scientific societies were included in the analysis. These documents covered a broad range of topics, including cardiomyopathies, atrial and ventricular arrhythmias, and specific clinical scenarios. The main characteristics of the included documents are summarized in Table 1.

**Table 1:** Overview of included guidelines and consensus documents

Document	Year	Society	Type	Main Focus	Relevance to antiarrhythmic therapy
AHA/ACC/HRS ventricular arrhythmias guideline	2017	AHA/ACC/HRS	Guideline	Ventricular arrhythmias, SCD prevention	Core AAD recommendations in VA
ESC ventricular arrhythmias guidelines	2022	ESC	Guideline	Ventricular arrhythmias and SCD	Updated AAD strategies and ICD integration
ESC cardiomyopathies guidelines	2023	ESC	Guideline	Cardiomyopathies	Phenotype-driven arrhythmia management
ESC atrial fibrillation guidelines	2024	ESC	Guideline	AF management	Rhythm control and AAD selection
ACC/AHA/HRS atrial fibrillation guideline	2023	AHA/ACC/HRS	Guideline	AF management	AAD use in structural heart disease
AHA/ACC HCM guideline	2024	AHA/ACC	Guideline	Hypertrophic cardiomyopathy	Disease-specific AAD strategies
HRS arrhythmogenic cardiomyopathy consensus	2019	HRS	Consensus	ACM/ARVC	AAD role in ventricular arrhythmias
EHRA electrical storm consensus	2023	EHRA	Consensus	Electrical storm	Acute AAD management
AHA scientific statement (AF in HFrEF)	2021	AHA	Scientific statement	AF + HF	Limits and role of AADs
HRS arrhythmias in athletes consensus	2024	HRS	Consensus	Athlete arrhythmias	AAD in special populations
EHRA antiarrhythmic drugs compendium	2025	EHRA	Consensus	AAD pharmacology	Drug-specific safety, contraindications, and practical prescribing considerations

*Summary of the clinical practice guidelines, expert consensus statements, scientific statements, and pharmacological compendia included in the qualitative synthesis, reported according to year of publication, issuing society, document type, main clinical focus, and relevance to antiarrhythmic drug therapy in cardiomyopathies

*AAD*, antiarrhythmic drug, *ACC* American College of Cardiology *ACM* arrhythmogenic cardiomyopathy, *AF* atrial fibrillation, *AHA* American Heart Association, *ARVC* arrhythmogenic right ventricular cardiomyopathy, *EHRA* European Heart Rhythm Association, *ESC* European Society of Cardiology, *HCM* hypertrophic cardiomyopathy, *HF *heart failure, *HFrEF* heart failure with reduced ejection fraction, *HRS* Heart Rhythm Society, *SCD* sudden cardiac death, *VA *ventricular arrhythmias

### General Principles

Across the included guidelines and consensus documents, AAD therapy in cardiomyopathies is consistently framed within a risk–benefit paradigm in which safety considerations prevail over efficacy. The presence of structural heart disease, myocardial fibrosis, and electrical instability significantly limits the use of several AADs, particularly class I, which are generally restricted or avoided in this setting due to their pro-arrhythmic potential [5].

As a result, therapeutic options are often restricted, and amiodarone emerges as the most widely applicable agent across different cardiomyopathy phenotypes, owing to its relative safety profile in structural heart disease despite its well-known extracardiac toxicity. Other agents, such as sotalol, may be considered in selected patients but require careful evaluation of pro-arrhythmic risk, particularly in the presence of left ventricular dysfunction or repolarization abnormalities [1].

Importantly, AAD therapy is rarely considered a definitive treatment strategy. Across guidelines, pharmacological therapy is frequently positioned as adjunctive to device-based or interventional approaches, including implantable cardioverter-defibrillators (ICDs) and catheter ablation, particularly in patients at high risk of ventricular arrhythmias [6].

A consistent theme across documents is the need for a phenotype-driven and individualized approach. The heterogeneity of cardiomyopathies, in terms of arrhythmic substrate, clinical presentation, and disease progression, precludes a uniform strategy, and therapeutic decisions should be tailored according to the underlying cardiomyopathy type, arrhythmia burden, and patient-specific risk factors. In this context, evidence supporting the use of AADs remains limited for cardiomyopathies other than hypertrophic and arrhythmogenic forms, and therapeutic decisions are largely extrapolated from broader populations with structural heart disease. Accordingly, class I agents are generally avoided, while beta-blockers, sotalol, and amiodarone represent the most commonly adopted pharmacological options [7].

Data on restrictive cardiomyopathies remain particularly scarce, and no specific antiarrhythmic recommendations are currently available; management is therefore extrapolated from broader structural heart disease populations. These overarching principles provide the framework for phenotype-specific strategies discussed below.

### Phenotype-Specific Recommendations

#### Hypertrophic Cardiomyopathy

Hypertrophic cardiomyopathy (HCM) is characterized by unexplained left ventricular hypertrophy, myocyte disarray, and interstitial fibrosis, which together create a complex arrhythmogenic substrate. In this setting, fibrosis and disarray favor re-entrant ventricular arrhythmias, while diastolic dysfunction and left atrial enlargement predispose to atrial fibrillation [8].

Accordingly, atrial fibrillation represents the most common arrhythmia in HCM and is associated with significant clinical consequences, including symptom burden and thromboembolic risk, whereas ventricular arrhythmias, although less frequent, remain the main determinant of sudden cardiac death in this population [4, 9].

In patients with HCM, medical therapy is indicated for both the management of left ventricular outflow tract obstruction (LVOTO) and arrhythmias, with several agents exerting overlapping hemodynamic and antiarrhythmic effects. However, current evidence does not support a role for AADs in the primary prevention of sudden cardiac death. In this context, according to contemporary ESC cardiomyopathy guidelines, beta-blockers and/or amiodarone may be considered in patients with ICDs who continue to experience symptomatic ventricular arrhythmias, paroxysmal atrial fibrillation, or recurrent appropriate shocks despite optimized medical therapy and device reprogramming [1].

Among beta-blockers, non-vasodilating agents, commonly used also for LVOTO management, are preferred, with metoprolol being among the most frequently employed [1]. Consistently, the EHRA compendium emphasizes beta-blockers as first-line therapy, given their beneficial effects on symptom control and potential reduction in ventricular arrhythmia burden, whereas other agents such as disopyramide and calcium channel blockers have not demonstrated prognostic benefit. Amiodarone is generally reserved for refractory cases or when other agents are not tolerated, given its lack of impact on sudden cardiac death and its long-term toxicity profile [10].

In the setting of atrial fibrillation, class IC agents such as flecainide and propafenone are contraindicated in the presence of significant left ventricular hypertrophy due to the risk of pro-arrhythmia [11]. According to AHA/ACC guidelines for HCM, amiodarone represents the most effective antiarrhythmic option for rhythm control, whereas dofetilide and sotalol may be considered with more modest efficacy. Disopyramide may be useful in selected patients with obstructive physiology because of its negative inotropic effect, particularly when symptom control or LVOTO reduction is also a therapeutic goal; in patients with atrial fibrillation, it should be combined with atrioventricular nodal blocking agents. Dronedarone appears to have limited efficacy, and no specific recommendations are currently provided regarding its use in this setting [8].

With regard to ventricular arrhythmias, beta-blockers, amiodarone, and, in selected cases, sotalol may be considered for symptomatic arrhythmia control; however, pharmacological therapy remains adjunctive, and ICD implantation continues to represent the cornerstone for sudden cardiac death prevention in high-risk patients [5, 7].

#### Dilated and Non-Dilated Left Ventricular Cardiomyopathies

Dilated cardiomyopathy and non-dilated left ventricular cardiomyopathy share a common arrhythmogenic substrate characterized by myocardial fibrosis, electrical remodeling, and conduction heterogeneity, resulting in a largely overlapping arrhythmic risk despite differences in ventricular geometry. The non-dilated phenotype encompasses a heterogeneous spectrum of conditions, including post-inflammatory myocardial remodeling and early or genetically determined forms characterized by myocardial fibrosis in the absence of overt ventricular dilation, which may differ in underlying pathophysiology but are currently managed according to similar arrhythmia-directed strategies.

Notably, most contemporary guidelines do not provide phenotype-specific recommendations for these entities, which include a wide range of underlying substrates. As a result, therapeutic strategies are largely extrapolated from broader populations with structural heart disease and heart failure, and are primarily guided by left ventricular ejection fraction. In this context, the lack of standardized definitions for terms such as “structural heart disease” and “minimal heart disease” further contributes to variability in treatment selection and interpretation of recommendations.

In this setting, structural and electrical remodeling promote slow conduction and re-entry mechanisms, predisposing to ventricular tachyarrhythmias, while increased filling pressures and atrial remodeling favor the development of atrial fibrillation. Consistently, the use of AADs is primarily limited by safety concerns, with class I agents generally avoided and beta-blockers, amiodarone, and, in selected cases, sotalol representing the main therapeutic options [10].

Regarding atrial fibrillation, in patients with reduced ejection fraction, the EHRA compendium recommends beta-blockers and amiodarone as first-line agents, whereas dronedarone should be avoided in the setting of decompensated heart failure [10]. Intravenous ibutilide may be used with caution for cardioversion, particularly in atrial flutter, while class IC agents and vernakalant are not recommended in patients with advanced heart failure [10, 11]. AHA/ACC/HRS guidelines support the use of dofetilide in patients with heart failure with reduced ejection fraction, whereas EHRA documents suggest caution due to its renal elimination and the associated risk of torsades de pointes, particularly in patients with impaired renal function [10, 12].

In patients with preserved ejection fraction, the EHRA compendium indicates that a broader range of antiarrhythmic options may be considered, including amiodarone, dofetilide, dronedarone, and sotalol [10]. Conversely, according to AHA/ACC guidelines, in the absence of significant structural heart disease or significant myocardial scar, class IC agents such as flecainide and propafenone may be used, highlighting the importance of accurate phenotypic characterization [13].

Regarding ventricular arrhythmias, according to the EHRA compendium, beta-blockers represent first-line therapy, also because of their established prognostic role in HFrEF populations, whereas amiodarone is considered the preferred second-line agent, particularly in patients who are not candidates for ICD implantation or who experience recurrent arrhythmias despite device therapy [10]. Sotalol may reduce ventricular arrhythmia burden and ICD therapies but is limited by its pro-arrhythmic potential and lack of survival benefit [10], and is not recommended in patients with severely reduced left ventricular function (LVEF < 20%) according to AHA/ACC/HRS guidelines [5]. In this context, the same guidelines indicate that beta-blockers and amiodarone, with sotalol in selected cases, may be considered in patients with non-ischemic cardiomyopathy and ICD who experience recurrent appropriate device therapies [5].

ESC ventricular arrhythmia guidelines recommend intensification of antiarrhythmic therapy, including the addition of amiodarone or substitution of beta-blockers with sotalol, in patients with dilated or non-dilated cardiomyopathy and recurrent symptomatic ventricular arrhythmias despite optimized therapy and device reprogramming [7].

Importantly, given the limited impact of pharmacological therapy on prognosis, with the exception of beta-blockers, risk stratification for sudden cardiac death and appropriate selection for ICD implantation remain central components of management in these patients, with AADs primarily serving an adjunctive role in reducing arrhythmic burden rather than improving survival.

Overall, AAD therapy in these phenotypes remains largely adjunctive, and clinical decision-making should integrate ventricular function, arrhythmic burden, myocardial fibrosis, and the potential risks associated with pharmacological therapy.

#### Arrhythmogenic Cardiomyopathy

Arrhythmogenic cardiomyopathy (ACM), including its classical right-dominant form (arrhythmogenic right ventricular cardiomyopathy, ARVC) as well as left-dominant and biventricular variants, is characterized by progressive fibro-fatty myocardial replacement, leading to electrical instability and a highly arrhythmogenic substrate. In this setting, myocardial scar formation and disruption of cell-to-cell coupling promote slow conduction and re-entry circuits, predisposing to ventricular tachyarrhythmias, which often occur early in the disease course and may precede overt structural abnormalities. Accordingly, ventricular arrhythmias represent the hallmark of the disease and are frequently the initial clinical manifestation, whereas atrial arrhythmias are less common and typically occur in more advanced stages, in association with disease progression and atrial remodeling. Although most specific recommendations refer to ARVC, they are discussed here within the broader ACM framework, which also includes left-dominant and biventricular variants.

According to ESC cardiomyopathy guidelines, beta-blockers are recommended as first-line therapy in patients with ARVC presenting with ventricular ectopy, non-sustained ventricular tachycardia (NSVT), or sustained ventricular tachycardia (VT). Amiodarone should be considered when beta-blockers fail to control arrhythmia-related symptoms, while flecainide should also be considered as second-line therapy in combination with beta-blockers [1].

Consistently, EHRA compendium and ESC ventricular arrhythmia guidelines identify beta-blockers as first-line therapy, while other AADs, including flecainide, sotalol, and amiodarone, have shown efficacy in suppressing ventricular arrhythmias mainly in small observational studies. In this context, combination therapy, particularly flecainide with beta-blockers or sotalol, has been associated with additional benefit in selected patients [7, 10].

AHA/ACC/HRS 2017 guidelines similarly recommend beta-blockers as first-line therapy but do not provide specific recommendations regarding second-line AAD use in this population [5]. In contrast, the 2019 h consensus document suggests a stepwise approach, with beta-blockers as first-line therapy, followed by amiodarone or sotalol as second-line options, and flecainide as third-line therapy, particularly in combination with beta-blockers in patients with ICD and preserved biventricular systolic function who remain symptomatic despite prior therapies [14].

Specific recommendations for the management of atrial fibrillation in patients with arrhythmogenic cardiomyopathy are lacking, and treatment is generally extrapolated from broader populations with structural heart disease.

### Special Clinical Scenarios

#### Electrical Storm

No phenotype-specific evidence is currently available for the management of electrical storm across cardiomyopathy subtypes. Current ESC ventricular arrhythmia guidelines and EHRA consensus on electrical storm recommend the use of beta-blockers, preferably non-selective agents, in combination with intravenous amiodarone in patients with electrical storm and structural heart disease, unless contraindicated [6, 7, 10].

According to the EHRA consensus on electrical storm, in patients with structural heart disease and hemodynamically tolerated ventricular tachycardia, procainamide may be considered, while lidocaine should be used with caution in the presence of impaired left ventricular function and has only modest efficacy outside the setting of acute ischemia. In addition, ranolazine has been shown in a randomized controlled trial to reduce the risk of recurrent ventricular tachycardia or ventricular fibrillation requiring ICD interventions, without a significant impact on mortality [6].

#### Athletes

In athletes with cardiomyopathies, no specific AAD recommendations are provided beyond disease-specific management. The 2024 h consensus on arrhythmias in the athlete emphasizes individualized risk assessment, shared decision-making, and careful consideration of the impact of pharmacological therapy on athletic performance and return-to-play decisions. In athletes with complex ventricular arrhythmias and underlying structural heart disease, management should therefore be guided by the underlying pathology, with catheter ablation, ICD therapy, or documentation of arrhythmia suppression during maximal exercise testing considered according to the clinical scenario [15].

Importantly, AAD therapy may influence exercise capacity and competitive performance in athletes. Beta-blockers, in particular, may impair physical performance and are restricted in certain precision sports, while sotalol may increase the risk of torsades de pointes, especially in the presence of resting bradycardia. In contrast, other antiarrhythmic agents, including amiodarone, class IC drugs, and calcium channel blockers, are not generally subject to sport-specific restrictions, although their use should remain individualized based on clinical context and safety considerations [10].

#### Pregnancy

Evidence on the use of antiarrhythmic drugs during pregnancy in patients with cardiomyopathies is limited, and management is largely guided by dedicated pregnancy guidelines and expert consensus rather than phenotype-specific data. According to the 2025 ESC guidelines on cardiovascular disease during pregnancy, therapeutic decisions should balance maternal arrhythmic risk and fetal safety, with preference for agents with established safety profiles and consideration of gestational pharmacokinetic changes [16].

Beta-blockers represent the cornerstone of arrhythmia management in several inherited and structural cardiac conditions during pregnancy, with the exception of atenolol, which should generally be avoided. Lipophilic agents such as labetalol, metoprolol, and propranolol are generally preferred, although dose adjustments may be required during mid and late pregnancy because of increased drug metabolism. Fetal growth, neonatal bradycardia, and hypoglycaemia should be monitored, particularly with prolonged exposure; during lactation, propranolol, metoprolol, and labetalol are associated with the lowest neonatal risk, whereas sotalol requires greater caution [16].

In pregnant patients with arrhythmogenic right ventricular cardiomyopathy, beta-blocker therapy should be continued or initiated when clinically indicated; flecainide and sotalol may be considered as second- and third-line options, respectively, with careful QTc and fetal growth monitoring, particularly when sotalol is used in the presence of reduced left ventricular ejection fraction [16]. In hypertrophic cardiomyopathy, beta-blockers remain first-line therapy for both arrhythmia control and left ventricular outflow tract obstruction, with verapamil as a second-line option [16].

Amiodarone is generally contraindicated during pregnancy because of the risk of fetal abnormalities, bradycardia, and thyroid dysfunction, and routine use is not recommended; however, a single dose may be considered in life-threatening emergencies such as ventricular tachycardia or cardiac arrest when maternal benefit outweighs fetal risk [16]. More broadly, ESC cardiomyopathy guidelines indicate that antiarrhythmic drugs should be continued or initiated only in the presence of a clear clinical indication and after individualized risk–benefit assessment [1].

## Discussion

### Principal Findings

This systematic review of contemporary guidelines and consensus documents highlights several consistent themes regarding the use of AADs in cardiomyopathies. First, across all phenotypes, AAD therapy is primarily guided by safety considerations rather than efficacy, reflecting the increased pro-arrhythmic risk associated with structural heart disease. As a result, the use of class I agents is generally restricted, while beta-blockers and amiodarone represent the most widely adopted pharmacological options.

Second, recommendations for AAD use remain only partially phenotype-specific, with the notable exception of hypertrophic and arrhythmogenic cardiomyopathies, where more tailored strategies are available. In contrast, in dilated and non-dilated left ventricular cardiomyopathies, therapeutic decisions are predominantly driven by left ventricular function rather than by the underlying disease substrate, highlighting a major gap in phenotype-oriented guidance.

Third, AAD therapy consistently emerges as an adjunctive strategy, primarily aimed at reducing arrhythmic burden and ICD therapies rather than improving survival. Accordingly, risk stratification for sudden cardiac death and appropriate ICD implantation remain the cornerstone of management across cardiomyopathies.

Finally, important gaps in evidence persist, particularly in less well-characterized phenotypes and in specific clinical scenarios such as pregnancy and competitive athletes, where recommendations are largely extrapolated from broader populations rather than supported by dedicated data.

**Fig. 2:**
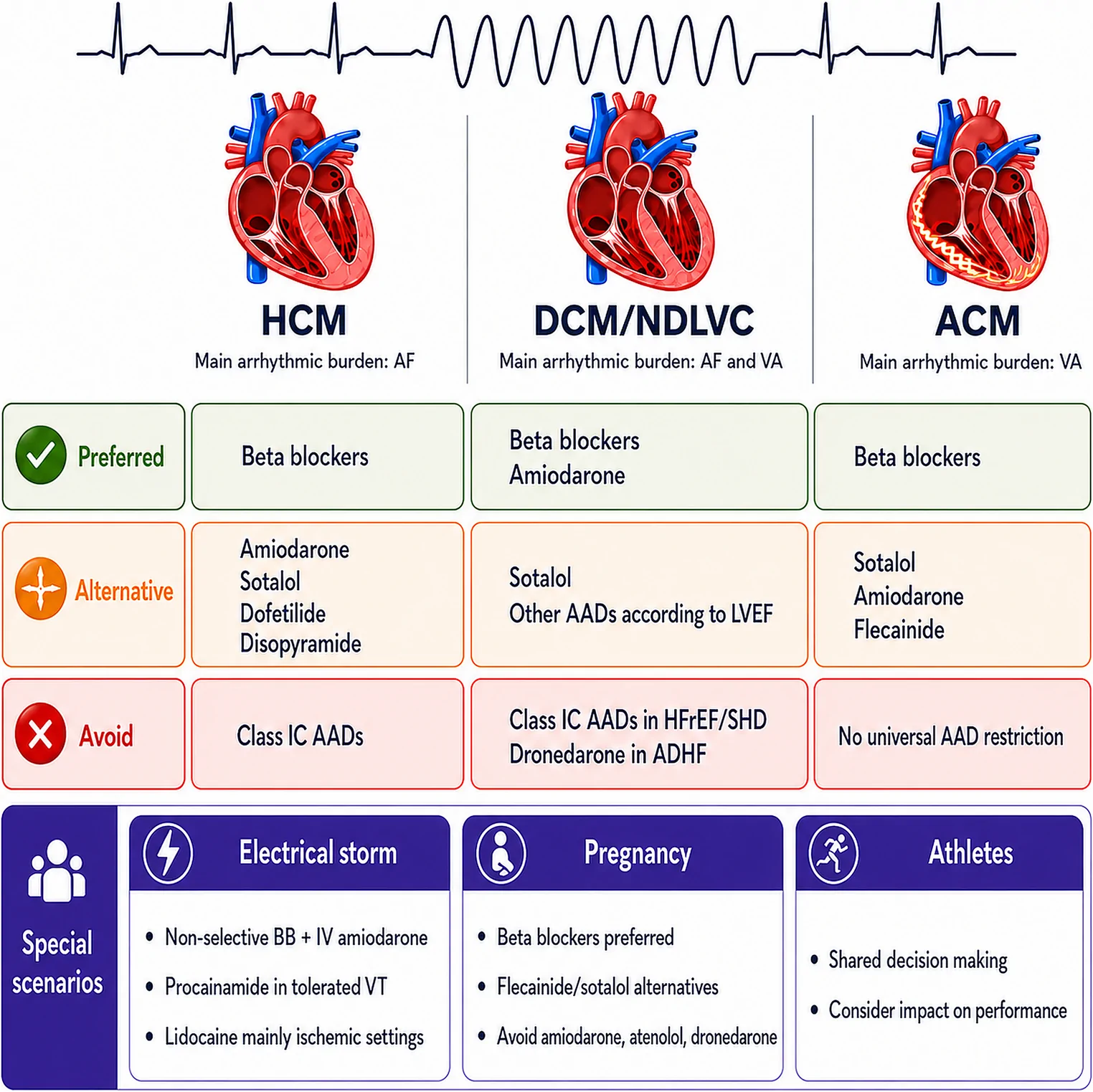
Antiarrhythmic drug therapy in cardiomyopathies: from phenotype to clinical context. Summary of preferred, alternative, and avoid/restricted antiarrhythmic drug strategies across major cardiomyopathy phenotypes and selected clinical scenarios, with indication of the predominant arrhythmic burden across phenotypes. AADs, antiarrhythmic drugs; ADHF, acute decompensated heart failure; BB, beta-blockers; HCM, hypertrophic cardiomyopathy; DCM, dilated cardiomyopathy; NDLVC, non-dilated left ventricular cardiomyopathy; ACM, arrhythmogenic cardiomyopathy; HFrEF, heart failure with reduced ejection fraction; SHD, structural heart disease; LVEF, left ventricular ejection fraction; VT, ventricular tachycardia. Created in BioRender. Palermi, A. (2026) https://BioRender.com/ze86y8j

A visual summary of the phenotype- and context-oriented antiarrhythmic strategies emerging from the included documents is provided in Fig. 2.

### Interpretation and Areas of Agreement/Discrepancy

The observed heterogeneity in recommendations across guidelines and consensus documents reflects several underlying limitations in the current evidence base and in the conceptual framework used to guide antiarrhythmic therapy in cardiomyopathies. A first major issue is the fragmentation of available guidance, with recommendations distributed across disease-specific documents (cardiomyopathies), arrhythmia-focused guidelines (atrial fibrillation and ventricular arrhythmias), and pharmacological consensus statements. This structural separation often results in partial overlap, inconsistencies, and variable emphasis on AAD use across documents.

A second key factor contributing to variability is the lack of standardized definitions for fundamental concepts such as “structural heart disease” and “minimal heart disease,” which are frequently used to guide AAD selection. These terms are inconsistently defined across guidelines and do not adequately capture the heterogeneity of cardiomyopathy phenotypes, particularly in entities such as non-dilated left ventricular cardiomyopathy, where myocardial fibrosis may be present in the absence of overt structural abnormalities.

Furthermore, the limited availability of randomized controlled trials specifically addressing AAD therapy in cardiomyopathies represents a major constraint. As a result, many recommendations are extrapolated from broader populations with heart failure or structural heart disease or are supported by small observational cohorts and expert consensus, rather than by phenotype-specific randomized evidence.

Despite these limitations, several areas of agreement can be identified across guidelines, including the prioritization of safety over efficacy, the central role of beta-blockers, the widespread use of amiodarone as the most versatile antiarrhythmic agent in structural heart disease, and the adjunctive role of pharmacological therapy relative to device-based and interventional strategies. Conversely, areas of discrepancy are most apparent in the positioning of specific agents, such as sotalol and class IC drugs, and in selected clinical scenarios requiring individualized risk–benefit assessment, reflecting differences in interpretation of limited evidence. These areas of agreement and disagreement are summarized in Table 2.

**Table 2:** Areas of agreement and discrepancy across guidelines

Domain	Areas of agreement	Areas of discrepancy / uncertainty	Clinical implications
General principles	Safety prioritized over efficacy; class I AADs generally avoided in structural heart disease; AADs as adjunctive therapy	Variable definitions of “structural heart disease” and “minimal heart disease”	Careful patient selection required; avoid extrapolation without phenotype consideration
Role of AADs	Beta-blockers as first-line therapy; amiodarone as most broadly applicable agent	Variable positioning of sotalol and other AADs across documents	Limited pharmacological options; individualized drug selection
HCM	Beta-blockers central; amiodarone effective for AF; ICD for SCD prevention	Variable role of sotalol, dofetilide, and disopyramide	AADs mainly for symptom and rhythm control; not for SCD prevention
DCM / NDLVC	AAD use guided by LVEF; beta-blockers and amiodarone preferred; class I avoided in reduced EF	Lack of phenotype-specific recommendations; reliance on extrapolation from HF/SHD	Management driven by ventricular function rather than phenotype
ACM / ARVC	Beta-blockers as first-line therapy	Heterogeneous recommendations for flecainide, sotalol, and amiodarone; consensus-based evidence	Significant variability in practice; need for individualized approach
Atrial fibrillation	EF-based AAD selection; amiodarone preferred in reduced EF	Differences in use of dofetilide, dronedarone, and class IC agents	Importance of integrating arrhythmia type with underlying substrate
Ventricular arrhythmias	Beta-blockers first-line; amiodarone second-line; ICD central	Role of sotalol and combination therapy varies	Pharmacological therapy mainly reduces arrhythmic burden
Electrical storm	Beta-blockers + IV amiodarone as standard approach	Role of procainamide, lidocaine, ranolazine based on limited data	Acute management largely consensus-driven
Special populations (athletes, pregnancy)	Individualized approach; preference for drugs with established safety profiles	Lack of phenotype-specific recommendations	Management extrapolated from general populations

*Summary of the main areas of agreement, discrepancy or uncertainty, and related clinical implications emerging from the comparative synthesis of guidelines and consensus documents on antiarrhythmic drug therapy in cardiomyopathies

*AADs* antiarrhythmic drugs, *ACM* arrhythmogenic cardiomyopathy, *AF* atrial fibrillation, *ARVC* arrhythmogenic right ventricular cardiomyopathy, *DCM* dilated cardiomyopathy, *EF* ejection fraction, *HCM* hypertrophic cardiomyopathy, *HF* heart failure, *ICD* implantable cardioverter-defibrillator, *IV* intravenous, *LVEF* left ventricular ejection fraction, *NDLVC* non-dilated left ventricular cardiomyopathy, *SCD* sudden cardiac death, *SHD* structural heart disease

### Limitations and Future Directions

This review should be interpreted in light of several limitations. First, the available evidence is inherently heterogeneous, as current recommendations derive from a combination of clinical practice guidelines, expert consensus documents, and pharmacological statements with different scopes and methodological approaches. Second, many recommendations are based on limited observational evidence or extrapolated from broader populations with structural heart disease or heart failure, reflecting the scarcity of randomized controlled trials specifically addressing AAD therapy in cardiomyopathies.

In addition, the systematic search strategy was primarily based on PubMed and focused on documents specifically addressing antiarrhythmic therapy in cardiomyopathies, while selected guidelines related to specific clinical scenarios, such as pregnancy, were included for contextual interpretation outside the formal selection process.

**Fig. 3:**
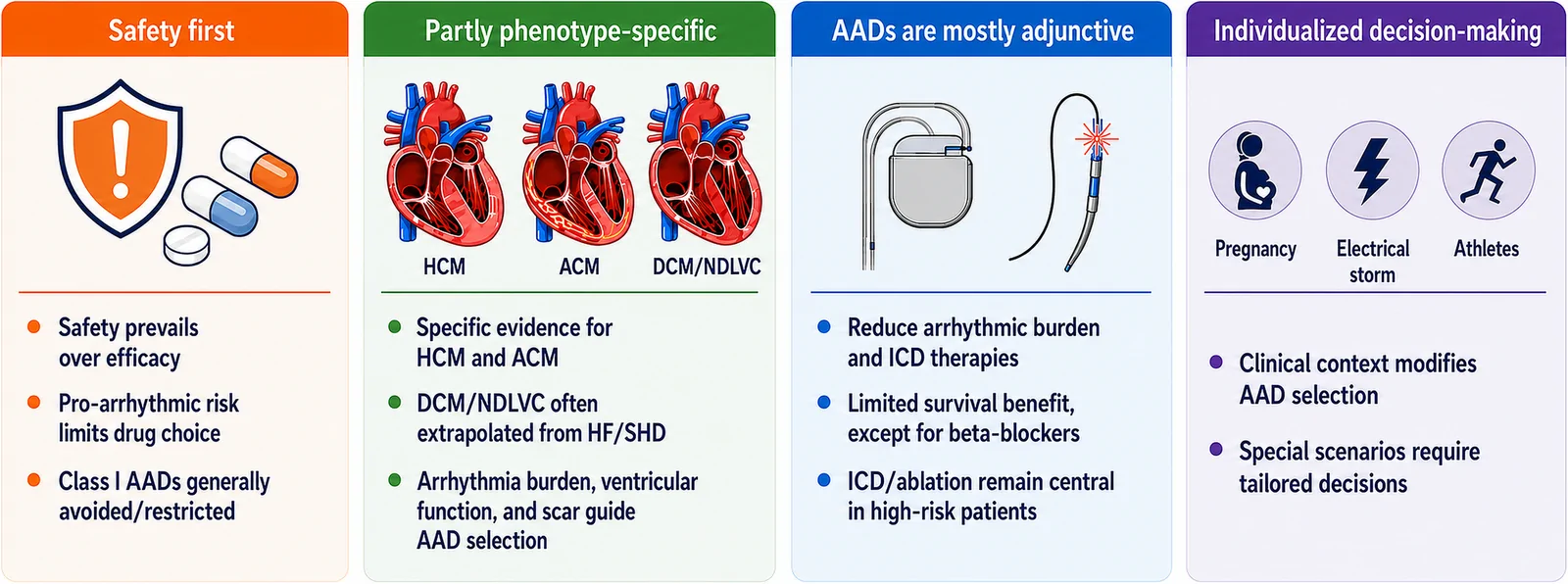
Central illustration: key messages on antiarrhythmic drug therapy in cardiomyopathies Summary of the main messages emerging from the comparative synthesis of guidelines and consensus documents. Antiarrhythmic drug therapy in cardiomyopathies is primarily safety-driven, only partly phenotype-specific, and mostly adjunctive to device-based and interventional strategies. Drug selection should consider arrhythmia burden, LVEF, myocardial scar, and clinical context, with special scenarios such as pregnancy, electrical storm, and athletic participation requiring tailored decisions. AADs, antiarrhythmic drugs; ACM, arrhythmogenic cardiomyopathy; DCM, dilated cardiomyopathy; HCM, hypertrophic cardiomyopathy; ICD, implantable cardioverter-defibrillator; LVEF, left ventricular ejection fraction; NDLVC, non-dilated left ventricular cardiomyopathy; SHD, structural heart disease. Created in BioRender. Palermi, A. (2026) https://BioRender.com/p62jlvj

Despite these limitations, the present review provides a clinically oriented synthesis of current recommendations and highlights major areas of agreement, discrepancy, and unmet need. Future studies specifically addressing phenotype-oriented antiarrhythmic strategies, particularly in non-dilated cardiomyopathies and other underrepresented phenotypes, are needed to improve the consistency and personalization of arrhythmia management in these patients. The key messages emerging from this comparative synthesis are summarized in Fig. 3.

## Conclusions

AAD therapy in cardiomyopathies remains a complex and predominantly safety-driven field, characterized by limited phenotype-specific evidence and substantial reliance on extrapolation from broader structural heart disease populations. Across contemporary guidelines and consensus documents, beta-blockers and amiodarone emerge as the most consistently adopted agents, whereas the role of other AADs varies according to ventricular function, arrhythmia type, and underlying substrate.

Overall, pharmacological therapy is primarily aimed at reducing arrhythmic burden and improving symptom control, while ICD and catheter ablation remain central components of management in high-risk patients.

The marked heterogeneity among cardiomyopathy phenotypes and the lack of dedicated randomized evidence highlight the need for more individualized and phenotype-oriented therapeutic strategies. Future studies specifically focused on antiarrhythmic therapy across different cardiomyopathy substrates are warranted to improve both consistency of recommendations and personalization of care.

## Key References

- Arbelo E, Protonotarios A, Gimeno JR, et al. 2023 ESC Guidelines for the management of cardiomyopathies. Eur Heart J. 2023;44:3503–3626.
  - ○ This guideline provides the most comprehensive contemporary framework for phenotype-oriented diagnosis and management of cardiomyopathies. It is particularly relevant because it integrates arrhythmia management within the broader clinical and genetic heterogeneity of cardiomyopathy phenotypes.
- Merino JL, Dan GA, Dobrev D, et al. Practical compendium of antiarrhythmic drugs: a clinical consensus statement of the European Heart Rhythm Association of the European Society of Cardiology. Europace. 2025;27.
  - ○ This recent EHRA consensus provides practical, drug-specific guidance on antiarrhythmic therapy, including contraindications, safety concerns, and prescribing considerations in structural heart disease. It is especially useful for translating guideline recommendations into clinical decision-making.

## Data Availability

No datasets were generated or analysed during the current study.

## **Abbreviations**

AAD: antiarrhythmic drug
ACC: American College of Cardiology
ACM: arrhythmogenic cardiomyopathy
ADHF: acute decompensated heart failure
AF: atrial fibrillation
AHA: American Heart Association
ARVC: arrhythmogenic right ventricular cardiomyopathy
BB: beta-blocker
DCM: dilated cardiomyopathy
EHRA: European Heart Rhythm Association
ESC: European Society of Cardiology
HCM: hypertrophic cardiomyopathy
HF: heart failure
HFrEF: heart failure with reduced ejection fraction
HRS: Heart Rhythm Society
ICD: implantable cardioverter-defibrillator
IV: intravenous
LGE: late gadolinium enhancement
LVEF: left ventricular ejection fraction
LVOTO: left ventricular outflow tract obstruction
NDLVC: non-dilated left ventricular cardiomyopathy
NSVT: non-sustained ventricular tachycardia
PRISMA: Preferred Reporting Items for Systematic Reviews and Meta-Analyses
RCM: restrictive cardiomyopathy
SCD: sudden cardiac death
SHD: structural heart disease
VA: ventricular arrhythmia
VF: ventricular fibrillation
VT: ventricular tachycardia
